# Toll-Like Receptor Ligands LPS and Poly (I:C) Exacerbate Airway Hyperresponsiveness in a Model of Airway Allergy in Mice, Independently of Inflammation

**DOI:** 10.1371/journal.pone.0104114

**Published:** 2014-08-04

**Authors:** Magnus Starkhammar, Olivia Larsson, Susanna Kumlien Georén, Marina Leino, Sven-Erik Dahlén, Mikael Adner, Lars-Olaf Cardell

**Affiliations:** 1 Divison of ENT Diseases, CLINTEC, Karolinska Institutet, Stockholm, Sweden; 2 Centre for Allergy Research, Karolinska Institutet, Stockholm, Sweden; 3 Institute for Environmental Medicine, Karolinska Institutet, Stockholm, Sweden; Murdoch University, Australia

## Abstract

It is well-established that bacterial and viral infections have an exacerbating effect on allergic asthma, particularly aggravating respiratory symptoms, such as airway hyperresponsiveness (AHR). The mechanism by which these infections alter AHR is unclear, but some studies suggest that Toll-like receptors (TLRs) play a role. In this study, we investigated the impact of TLR3 and TLR4 ligands on AHR and airway inflammation in a model of pre-established allergic inflammation. Female BALB/c mice were sensitised and challenged intranasally (i.n.) with either PBS or ovalbumin (OVA) and subsequently i.n. challenged with poly (I:C) (TLR3) or LPS (TLR4) for four consecutive days. The response to methacholine was measured *in vivo*; cellular and inflammatory mediators were measured in blood, lung tissue and broncheoalveolar lavage fluid (BALF). OVA challenge resulted in an increase in AHR to methacholine, as well as increased airway eosinophilia and TH2 cytokine production. Subsequent challenge with TLR agonists resulted in a significant increase in AHR, but decreased TLR-specific cellular inflammation and production of immune mediators. Particularly evident was a decline in LPS-induced neutrophilia and neutrophil-associated cytokines following LPS and poly (I:C) treatment. The present data indicates that TLRs may play a pivotal role in AHR in response to microbial infection in allergic lung inflammation. These data also demonstrate that aggravated AHR occurs in the absence of an exacerbation in airway inflammation and that allergic inflammation impedes a subsequent inflammatory response to TLRs. These results may parallel clinical signs of microbial asthma exacerbation, including an extended duration of illness and increased respiratory symptoms.

## Introduction

It is a well-established clinical phenomenon that viral and bacterial infections are primary risk factors for acute asthma exacerbations [1], [2]. Viral infections, such as respiratory syncytial virus (RSV) and human rhinovirus (HRV), are the most predominant forms of asthma exacerbation, believed to cause almost 50% of exacerbations in adults [3]. Bacterial infections, such as *Streptococcus pneumoniae, Haemophilius influenzae* and *Moraxella cattarhalis* are also present during asthma exacerbations and have been shown to be both triggers of the exacerbation or play an opportunistic role following viral infection [4]. Moreover, microbial exacerbations have been shown not only to extend the period of illness, but also increase the respiratory symptoms, such as airway hyperresponsiveness (AHR) [5], [6], [7]. Despite the breadth of epidemiological studies in this area, the mechanisms behind these phenomena are still poorly understood. However, recent data suggest that pattern recognition receptors (PRRs) of the innate immune system may play a pivotal role [8], [9], [10].

PRRs make up a wide range of molecules able to recognise pathogen-associated molecular patterns (PAMPs), conserved components of infectious pathogens, such as viruses or bacteria. The Toll-like receptors (TLRs) are a widely studied subset of PRRs, of which at least 10 are now recognised in humans and 13 in mice [11]. TLR3, which recognises viral double-stranded RNA (dsRNA), and TLR4, which recognises lipopolysaccharide (LPS), play a key role in the initial innate response against viruses and bacteria, respectively, and are vital players in the development of an adaptive immune response [12], [13]. Two key pathogens known to be involved in asthma exacerbation, namely RSV [3] and *S. pneumonia* [4], are recognised by TLR3 [14] and TLR4 [15] respectively, suggesting these PRRs may play a role in exacerbation. Indeed, we and several other groups have demonstrated that TLRs in both immune and non-immune cells are associated with pathogenesis of airway allergy, being implicated in development of asthma, exacerbation and AHR [8], [16], [17], [18], [19].

In a previous study, we demonstrated that four consecutive days of treatment of mice with the TLR3 ligand poly (I:C), a synthetic analogue of dsRNA, or the TLR4 ligand LPS, resulted in prominent AHR, but disparate airway inflammatory patterns [19]. Using the same set-up, this study instead aimed to investigate the impact of microbial infection on a pre-established allergic inflammatory event in the lung, by investigating AHR and inflammation. TLR3 and TLR4 ligands were given on four consecutive days following establishment of an allergic response against OVA. AHR was assessed by measuring reactivity to methacholine *in vivo* and inflammation was characterised through measurement of leukocytes in blood, tissue and broncheoalvoelar lavage fluid (BALF) and cytokines in BALF. We hypothesised that both ligands would exacerbate AHR and this may be associated with an increased inflammatory response. Interestingly, it was found that TLR ligand stimulation did indeed increase AHR above that induced by OVA-treatment alone, but resulted in no further change to inflammation, suggesting a dissociation between airway inflammation and AHR.

## Materials and Methods

### Animals

Adult female BALB/c mice (6–8 weeks) were obtained from Charles River (Sulzfeld, Germany). They were housed in groups in plastic cages with adsorbent bedding in a temperature and light-dark cycle (12 h:12 h) controlled room. Food and water were available *ad libitum*. All animal procedures were performed in an experimental animal laboratory; all surgical techniques were performed under sodium pentobarbital anaesthesia to minimise animal suffering. If any animals showed signs of ill health prior to the experimental endpoint (according to guidelines issued by Karolinska Institutet), the animals were sacrificed by cervical dislocation. At the end of each experimental time point, animals were sacrificed by cervical dislocation. All animal procedures were approved by the local ethics committee at Karolinska Institutet (Stockholm norra djurförsöksetiska nämnd; ethical permit numbers: 152/06 and 153/11).

### Treatment Protocol

Mice were sensitised and challenged with OVA and treated with TLR ligands, as previously described [8]. Briefly, mice were sensitized by an intraperitoneal (i.p.) injection of 10 µg ovalbumin (OVA, grade II, Sigma-Aldrich, St. Louis, MO, USA) and 1 mg Al (OH)_3_ (Sigma-Aldrich) suspended in 200 µl PBS on days 1 and 8. Mice were subsequently challenged intranasally (i.n.) with 50 µg OVA (suspended in 20 µl PBS) under isoflurane anaesthesia, on days 15, 16 and 17. On days 18–21, mice were challenged i.n. with 20 µl 0.1 mg·ml^−1^ LPS from *Escherichia coli* (0127:B8, Sigma-Aldrich) or 20 µl 1 mg·ml^−1^ poly (I:C) (Sigma-Aldrich), under isoflurane anaesthesia. Lung mechanics, as well as terminal collection of broncheoalveolar lavage fluid (BALF), blood and tissue were performed on day 22, 24 h after the final i.n. challenge. Thirteen to 14 animals were allocated for measurement of lung mechanics and collection of BALF; six animals were allocated for collection of blood and tissue. The number of mice per group was chosen based on previously carried out experiments. Mice were randomly allocated to each treatment group.

### Lung Mechanics

Mice were ventilated with a flexiVent animal ventilator (Scireq, Montreal, Canada), as previously described [8], [19]. Following anaesthesia with sodium pentobarbital (90 mg·kg^−1^) and tracheotomy, animals were placed on a heating pad (37°C) and connected to the ventilator via an 18-gauge cannula. Once ventilated, a bilateral thoracotomy was performed to equalize pleural and atmospheric pressure, as well as to exclude any chest wall contributions to the mechanics. Mice were ventilated at a frequency of 2.5 Hz in a quasi-sinusoidal fashion wherein the pressure waveform was sinusoidal during inflation. The tidal volume was set at 12 ml·kg^−1^ body weight and the positive end-expiratory pressure (PEEP) to 3 cm H_2_O. An intravenous (i.v.) catheter was inserted into the tail vein for induction of AHR.

Four sigh manoeuvres of three times the tidal volume were performed to stabilize the baseline lung resistance (R_L_). After a five minute resting period, AHR was induced via i.v. injections of increasing doses of Acetyl-β-methylcholine (MCh) (Sigma-Aldrich) (0.01, 0.03, 0.1, 0.3, 1 and 3 mg·kg^−1^·body weight). R_L_ was measured by assuming a single-compartment linear model and multiple linear regressions. Changes in reactivity and sensitivity were assessed using non-linear regression analysis to calculate the maximum responses (R_Lmax_) and the effective dose for half the maximal response (EC_50_).

### Broncheoalveolar Lavage (BAL) and Differential Cell Counts

BAL was performed immediately following lung function measurements. BAL fluid (BALF) was collected by inserting and excising 1 ml PBS containing 0.6 M ethylendiaminetetraacetic acid (EDTA, Sigma-Aldrich) into the lungs three times. The fluid was centrifuged at 4°C for 10 minutes at 1200 rpm and supernatant was stored at −80°C until further use. To lyse red blood cells, the pellet was incubated with lysis buffer [150 mM NH_4_Cl, 10 mM KHCO_3_, 0.1 mM EDTA, pH 7.2] for two minutes and subsequently washed in PBS. Total cell number was counted using a haemocytometer to calculate cells·ml^−1^ BALF. Differential cell counts were performed on May-Grünwald/Giemsa stained cytospins. A minimum of 300 cells were counted, in a blinded manner.

### Flow Cytometry

Inflammatory cells in blood and lung tissue were analysed on an LSRFortessa Analyser Flow Cytometer (BD, San Jose, USA). Lung tissue was homogenized and placed through a 100 µm cell strainer (BD Falcon) and subsequently diluted to a concentration of 1000000 cells·ml^−1^. 1 ml cell suspension and 50 µl blood was stained with antibodies to detect leukocyte populations (Table 1). Leukocytes were gated based on expression of CD45; neutrophils were identified as Ly6G^+^Ly6C^+^CD11b^+^SiglecF^−^ and eosinophils were identified as CD11b^+^SiglecF^+^Ly6G^−^. Cells were back-gated onto a FSC/SSC plot to confirm the cells as granulocytes. Data was analysed on FlowJo Analysis Software (TreeStar Inc., Ashland, USA). Total cells per µl blood were determined by counting total events in a fixed volume analysed by the flow cytometer and comparing back to the total volume stained. Total cells per mg tissue were determined by counting the total cells recovered following homogenisation and dividing by the mg tissue recovered.

**Table 1:** Antibodies used in flow cytometry for analysis of leukocytes in lung tissue and blood.

Marker	Fluorophore	Clone	Isotype	Supplier	Concentration
**CD45**	V500	30-F11	Rat IgG2b κ	BD Biosciences	0.4 µg/10^6^ cells
**Ly6G**	Pacific Blue	1A8	Rat IgG2a κ	BD Biosciences	0.4 µg/10^6^ cells
**CD11b**	PerCP-Cy5.5	M1/70	Rat IgG2b κ	BD Biosciences	0.4 µg/10^6^ cells
**Ly6C**	Pe-Cy7	AL-21	Rat IgM κ	BD Biosciences	0.6 µg/10^6^ cells
**CD3**	Pacific Blue	17A2	Rat IgG2a κ	BD Biosciences	0.4 µg/10^6^ cells
**CD4**	PerCP	RM4-5	Rat IgG2a κ	BD Biosciences	0.4 µg/10^6^ cells
**Siglec-F**	PE-CF594	E50-2440	Rat IgG2a	BD Biosciences	0.4 µg/10^6^ cells

### Measurement of Inflammatory Mediators

Cytokines in BALF were measured using the Cytokine Mouse 20-Plex Panel and RANTES Mouse Singleplex Bead Kit (Invitrogen, San Diego, USA), according to the manufacturer’s instructions. Briefly, samples and standards were diluted with assay diluent and applied to spectrally encoded beads. Following incubation, beads were washed and mixed with specific biotinylated detector antibodies. Streptevadin-R-phycoerythrin (RPE) was subsequently added to label immune complex formation on the beads. Cytokine concentrations were determined by measuring spectral properties of the beads using the Bioplex System (BioRad Laboratories, Hercules, CA, USA).

### Statistical Analysis

Data was analysed using GraphPad Prism Software (San Deigo, CA, USA). Results are presented as mean ± SEM and n is equal to the number of subjects (mice). The impact of OVA and TLR-ligand challenge on airway resistance (R_Lmax_ and EC_50_) was analysed using a one-way ANOVA followed by a Tukey multiple comparison post-test. Blood, tissue and BALF cell number and cytokine levels were analysed using a two-way ANOVA followed by a Bonferroni multiple comparison post-test. A p-value of 0.05 or less was considered to be statistically significant.

## Results

### Airway Hyperresponsiveness

**Figure 1:**
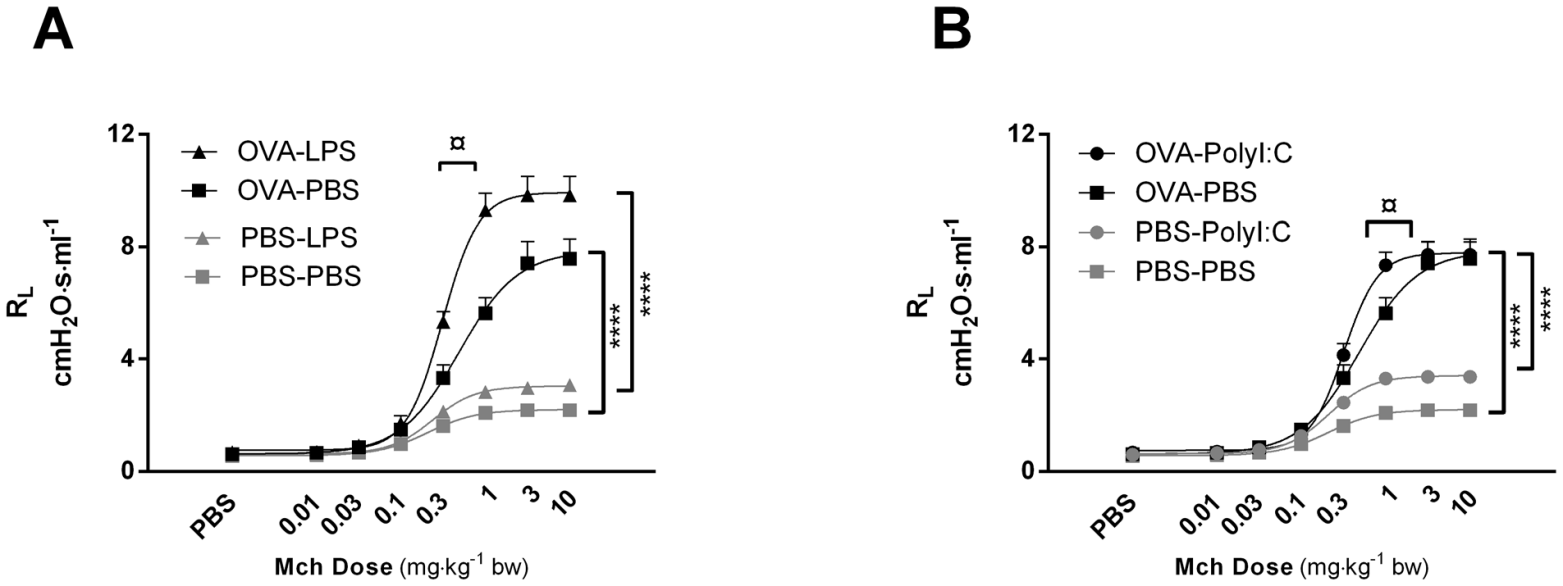
Airway hyperresponsiveness in OVA-sensitised and challenged mice treated with LPS or poly (I:C). All animals were sensitised i.p. with OVA/Al (OH)_3_ and subsequently challenged i.n. with PBS or OVA (3 days) and PBS, LPS or Poly (I:C) (4 days). 24 hrs after the final challenge, changes in lung resistance (R_L_) in response to increasing doses of methacholine (MCh) was measured using the flexiVent animal respirator. Data is represented as mean resistance (R_L_) ± SEM. (A) Animals were treated with PBS or LPS for 4 days following OVA or PBS treatment. (B) Animals were treated with PBS or Poly (I:C) for 4 days following OVA or PBS treatment. ¤ p<0.05 comparing EC_50_ values between OVA-PBS and OVA-LPS or OVA-PBS and OVA-Poly (I:C) using one-way ANOVA followed by a Tukey multiple-comparison post-test. ****p<0.0001 comparing R_Lmax_ of OVA and PBS challenged groups (OVA-PBS vs. PBS-PBS; OVA-LPS vs. PBS-LPS; OVA-Poly (I:C) vs. PBS-Poly (I:C)) using one-way ANOVA followed by a Tukey multiple-comparison post-test. n = 13–16 animals per group.

To investigate the impact of TLR3 and TLR4 stimulation on airway function in animals with pre-established allergic inflammation, a dose-response to MCh was carried out in challenged animals with and without prior lung inflammation. As expected, animals sensitized to and challenged with OVA (henceforth referred to as “OVA animals” for lack of complication) displayed an increase in R_L_ as compared to vehicle-challenged controls (“PBS animals”), with a significant increase in the maximal response (R_Lmax_) to MCh (PBS-PBS: 2.21±0.21 cmH_2_O·s·ml^−1^ vs. OVA-PBS: 8.83±1.01 cmH_2_O·s·ml^−1^; p<0.0001) (Figure 1). Similarly, OVA animals treated with LPS showed significant increases in R_Lmax_ (PBS-LPS: 3.28±0.36 cmH_2_O·s·ml^−1^ vs. OVA-LPS: 9.88±0.70 cmH_2_O·s·ml^−1^; p<0.0001), as compared to LPS-treated PBS animals (Figure 1A) and a significant difference was seen between OVA and PBS animals treated with poly (I:C) (PBS-poly (I:C): 3.41±0.11 cmH_2_O·s·ml^−1^ vs. OVA-poly (I:C): 7.8±0.48 cmH_2_O·s·ml^−1^, p<0.0001) (Figure 1B).

More pertinent to this study, OVA animals treated with LPS showed a significant increase in potency to MCh as compared to non-treated OVA animals (EC_50_ values: OVA-LPS: 0.305±0.031 vs. OVA-PBS: 0.511±0.86, p = 0.021) (Figure 1A). Similarly, OVA animals treated with poly (I:C) also demonstrated a significant change in potency as compared to non-treated OVA animals (EC_50_ values: OVA-poly (I:C): 0.332±0.026 vs. OVA-PBS: 0.511±0.86, p = 0.040) (Figure 1B).

### Cellular Inflammation

**Figure 2:**
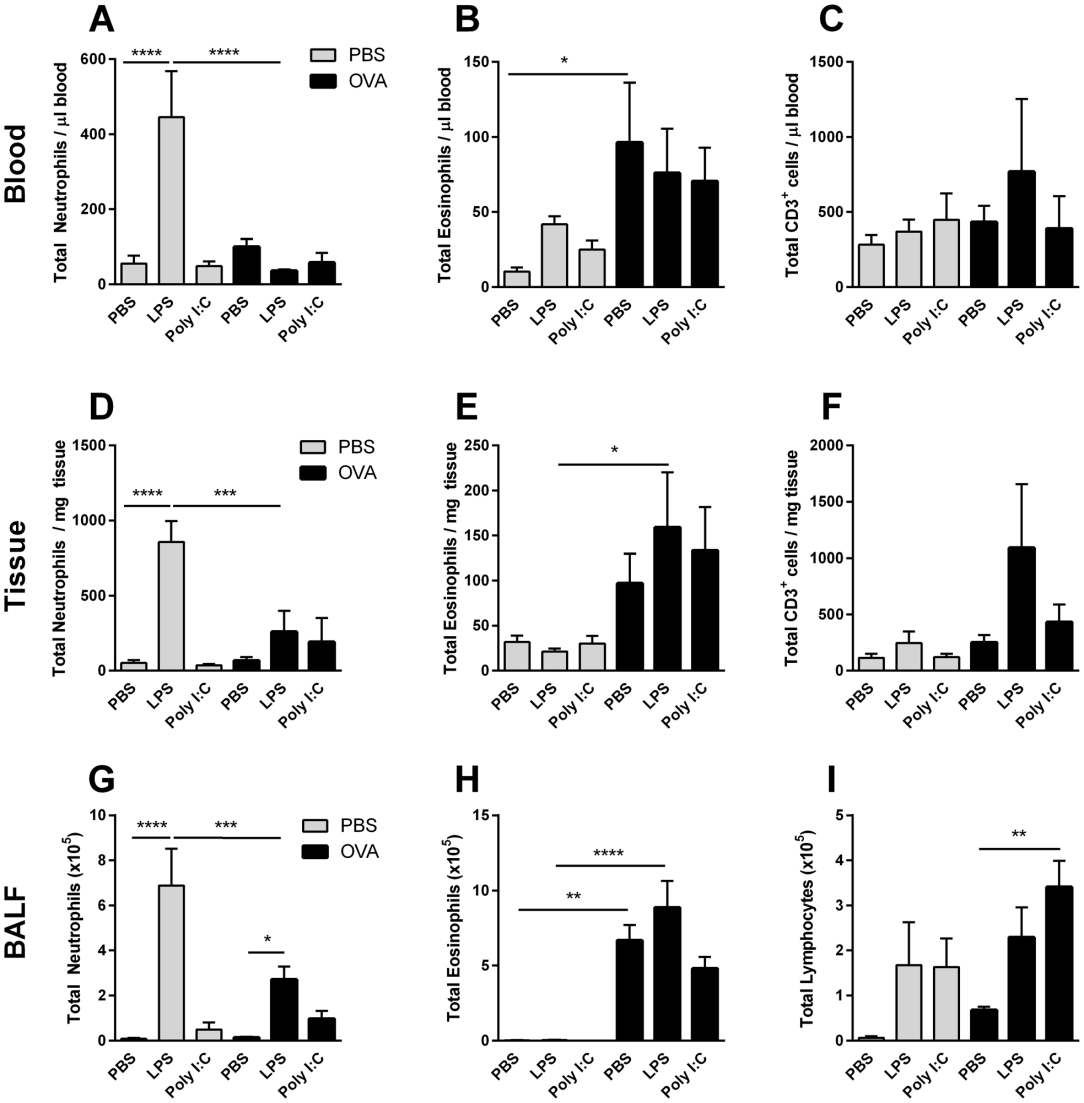
Inflammatory cells in blood (A–C), tissue (D–F) and BALF (G–I) following TLR ligand challenge in OVA-sensitised mice. All animals were sensitised i.p. with OVA/Al (OH)_3_ and subsequently challenged i.n. with PBS (grey bars) or OVA (black bars) (3 days) and PBS, LPS or Poly (I:C) (4 days). 24 hrs after the final challenge, blood, tissue and BALF were collected. Neutrophils (A, D), eosinophils (B, E) and CD3^+^ cells (C, F) in blood and tissue were determined using flow cytometry; a differential cell count was used to determine neutrophil (G), eosinophil (H) and lymphocyte (I) number in BALF. Data is represented as mean ± SEM. Data was analysed using a two-way ANOVA followed by a Bonferroni multiple comparison post-test *p<0.05, **p<0.01, ***p<0.001, ****p<0.0001. n = 6 (blood, tissue) or 14 (BALF) animals per group.

To investigate the impact of pre-established allergic inflammation on the inflammatory response to LPS and poly (I:C), cellular inflammation was analysed in blood, lung tissue and BALF (Figure 2). As expected, OVA sensitisation and challenge was associated with a significant rise in eosinophils in blood (Figure 2B), tissue (Figure 2E) and BALF (Figure 2H) in all animals (blood: p = 0.045; tissue: p<0.001; BALF: p<0.0001).

In blood, tissue and BALF of PBS animals, LPS treatment was associated with a significant elevation in neutrophil numbers (blood: p<0.0001; tissue: p<0.0001; BALF: p<0.0001), as compared to vehicle-treated animals (Figure 2A, D, G). However, a rise in neutrophil numbers after LPS treatment in OVA-animals was apparent only in BALF (p = 0.014) (Figure 2G). In fact, LPS-treated PBS animals demonstrated significantly higher neutrophil numbers in blood, tissue and BALF (blood: p<0.0001, tissue: p<0.001, BALF: p<0.001), as compared to LPS-treated, OVA-challenged animals. Poly (I:C) treatment was not associated with significant changes in neutrophil numbers in any of the compartments investigated (Figure 2A, D, G).

Poly (I:C) was associated with elevated levels of lymphocytes in BALF (p = 0.009) (Figure 2I). A significant elevation in lymphocytes in BALF was particularly evident in OVA animals treated with poly (I:C) (p = 0.008). Total numbers of CD3^+^ lymphocytes in blood (Figure 2C) and tissue (Figure 2F) were not significantly altered; however, an altered proportion in CD3^+^ cells between LPS− and poly (I:C)-treated animals was evident, with a significant increase in CD3^+^ cells following poly (I:C), but not LPS challenge (Figure S1).

### Inflammatory Mediators

**Figure 3:**
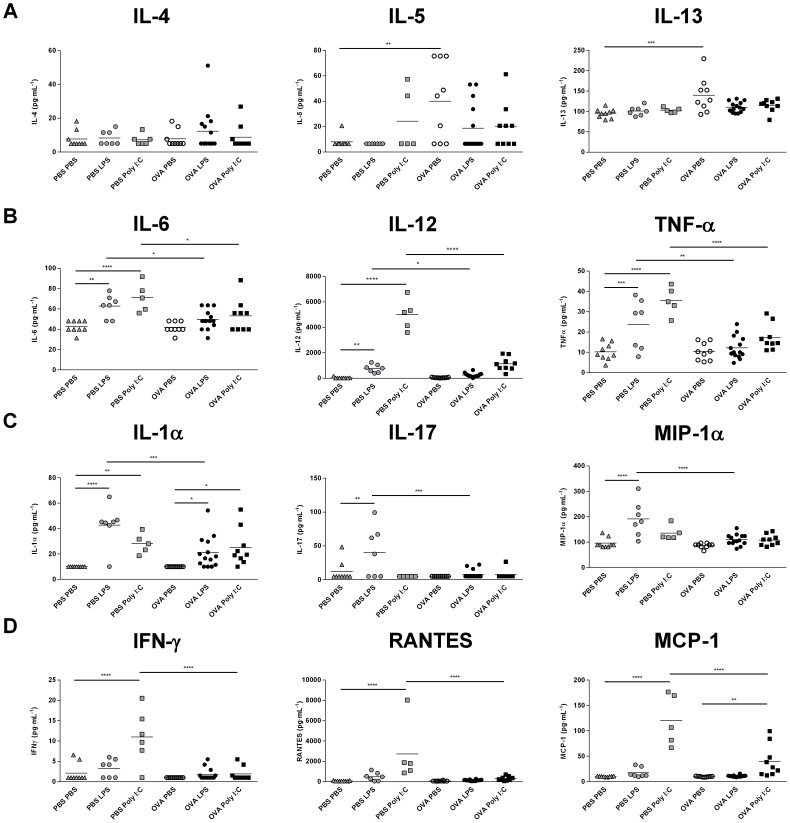
Inflammatory mediators in BALF following TLR ligand challenge in OVA-sensitised mice. All animals were sensitised i.p. with OVA/Al (OH)_3_ and subsequently challenged i.n. with PBS or OVA (3 days) and PBS, LPS or Poly (I:C) (4 days). 24 hrs after the final challenge, BALF was extracted and cytokine levels were measured using the Cytokine Mouse 20-Plex Panel and the RANTES Mouse Singleplex Bead Kit. Data is represented as mean ± SEM. Data was analysed using a two-way ANOVA followed by a Bonferroni multiple comparison post-test *p<0.05, **p<0.01, ***p<0.001, ****p<0.0001. n = 6–14 animals per group.

To further investigate the impact of pre-established lung inflammation on the inflammatory response to TLR3 and TLR4 ligands, inflammatory cytokines and chemokines were measured in BALF (Figure 3). As expected, OVA sensitisation and challenge was associated with a significant upregulation in the TH2-cytokines IL-5 (p = 0.019) and IL-13 (p<0.001) in BALF, but had no effect on levels of IL-4 (p = 0.389) at the time-point measured (Figure 3A). In PBS animals, LPS challenge was associated with a significant increase in innate inflammatory cytokines IL-6 (p = 0.002), IL-12 (p = 0.006), TNF-α (p<0.001), (Figure 3B), IL-17 (p = 0.002), and IL-1α (p<0.0001) (Figure 3C), as well as the chemokine macrophage inflammatory protein 1α (MIP-1α) (p<0.0001) (Figure 3C). Poly (I:C) challenge was similarly associated with a significant increase in IL-6 (p<0.0001), IL-12 (p<0.0001) and TNF-α (p<0.0001) (Figure 3B) in PBS animals, and was additionally associated with a significant upregulation in the anti-viral mediator IFN-γ (p<0.0001), as well as the chemokines RANTES (regulated upon activation, normal T-cell expression and secreted) (p<0.0001) and monocyte chemotactic protein 1 (MCP-1) (p<0.0001) (Figure 3D).

Comparatively, IL-1α (OVA-PBS vs. OVA-LPS: p = 0.041; OVA-PBS vs. OVA-Poly (I:C): p = 0.010) (Figure 3C) and MCP-1 (OVA-PBS vs. OVA-Poly (I:C): p = 0.007) (Figure 3D) were the only cytokines upregulated following TLR stimulation in OVA-sensitised and challenged animals. A prior OVA challenge was found to have a significant effect on TLR3/4-induced production of all inflammatory mediators measured (IL-6: p = 0.001; IL-12: p<0.0001; TNF-α: p<0.0001; IL-1α: p = 0.012; IL-17: p = 0.009; MIP-1α: p<0.0001; IFN-γ: p<0.0001; RANTES: p<0.001; MCP-1: p<0.0001). OVA-challenge was associated with a significant decline in LPS-induced IL-6 (p = 0.032), IL-12 (p = 0.043), TNF-α (p = 0.002) (Figure 3B), IL-1α (p<0.001), IL-17 (p = 0.0001) and MIP-1α (p<0.0001) (Figure 3C), as well as a significant reduction in poly (I:C)-induced IL-6 (p = 0.012), IL-12 (p<0.0001), TNF-α (p<0.0001) (Figure 3B), IFN-γ (p<0.0001), RANTES (p<0.0001) and MCP-1 (p<0.0001) (Figure 3D).

### Inflammatory Profile

**Figure 4:**
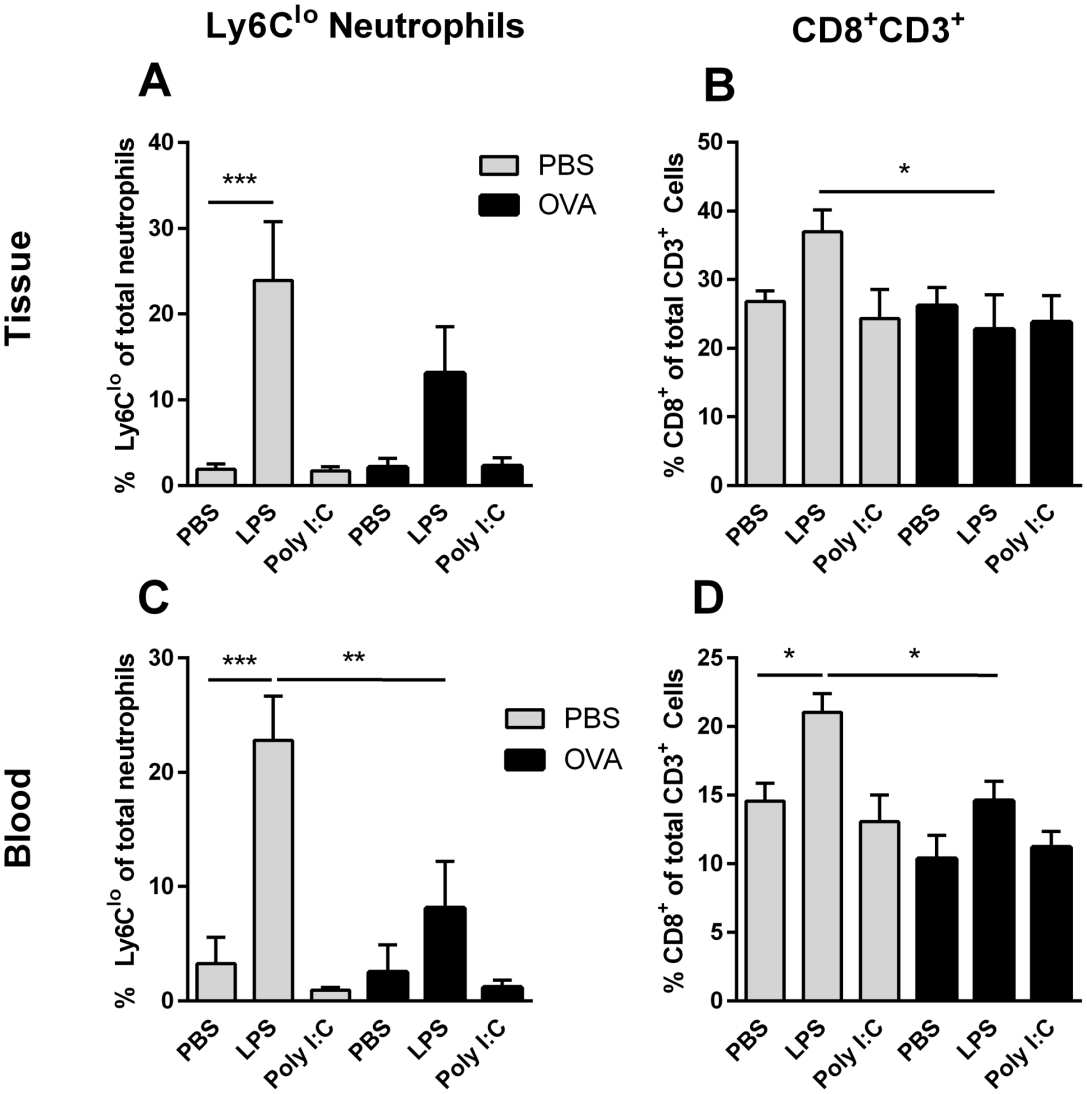
Neutrophil and T-lymphocyte populations in blood and tissue following TLR ligand challenge in OVA-sensitised mice. All animals were sensitised i.p. with OVA/Al (OH)_3_ and subsequently challenged i.n. with PBS (grey bars) or OVA (black bars) (3 days) and PBS, LPS or Poly (I:C) (4 days). 24 hrs after the final challenge, blood (C, D) and lung tissue (A, B) were collected and percent of Ly6C^lo^ neutrophils (A, C) and CD8^+^ CD3^+^ lymphocytes (B, D) were measured by flow cytometry. Data is represented as mean percent ± SEM. Data was analysed using a two-way ANOVA followed by a Bonferroni multiple comparison post-test *p<0.05, **p<0.01, ***p<0.001; n = 6 per animals per group.

As OVA-induced allergic inflammation was found to prevent aspects of cellular inflammation and production of inflammatory mediators induced by TLR3 and TLR4 stimulation, the inflammatory profile was more closely investigated by flow cytometry, via analysis of neutrophil and T-lymphocyte populations, particularly CD8^+^ T-lymphocytes and a population of Ly6C^lo^ neutrophils (Figure 4). In PBS animals, LPS challenge was associated with a significant upregulation in percent of Ly6C^lo^ neutrophils in tissue (p<0.001) (Figure 4A) and blood (p = 0.0001) (Figure 4C). Comparatively no change in percent of Ly6C^lo^ neutrophils was apparent following LPS-challenge of OVA animals (Figure 4A, C). Poly (I:C) was not associated with a change in Ly6C^lo^ neutrophils in PBS or OVA animals (Figure 4A, C).

There was no effect of TLR ligand treatment on percent of CD3^+^CD4^+^ in tissue or blood (Figure S2). LPS challenge was, however, associated with a significant upregulation in percent of CD3^+^CD8^+^ cells in blood of PBS animals (p = 0.013). However, no significant change was seen following LPS challenge in blood of OVA animals (Figure 4D). In fact, following LPS-challenge, percent of CD3^+^CD8^+^ cells was significantly lower in both tissue (p = 0.031) (Figure 4B) and blood (p = 0.013) (Figure 4D) of OVA animals, as compared to PBS animals. No significant change in percent of CD3^+^CD8^+^ cells was seen in blood or tissue following poly (I:C) challenge (Figure 4B, D).

## Discussion

The aim of the current study was to mimic a microbial infection by administering TLR3 or TLR4 agonists in a model of pre-established allergic lung inflammation. Compared to ligand stimulation in non-allergic animals, AHR in response to LPS and poly (I:C) was heightened on a background of allergy in the lung. In addition, both ligands significantly elevated AHR beyond that induced by OVA-sensitisation and treatment itself, findings that may correlate with the clinical picture of severe bacterial and viral exacerbations in asthmatics. An elevation in allergic inflammation was also apparent following OVA-treatment, but the inflammatory response to TLR ligand stimulation, particularly LPS, was dampened on a background of allergic inflammation.

### Establishment of a model of asthma exacerbation

Sensitisation and subsequent intranasal challenge with OVA is a well-established model to induce allergic lung inflammation in rodents [20], [21]. In this study, our protocol was shown to mimic prototypical characteristics of clinical atopic asthma, namely blood, tissue and BALF eosinophilia, an elevation of TH2 cytokines IL-5 and IL-13 in BALF, and prominent AHR, as shown by a heightened reactivity to methacholine. This effect was sustained 5 days after the final OVA challenge, as previously shown [8]. Using this model, we subsequently investigated the impact of four consecutive days of LPS or poly (I:C) treatment on AHR and inflammation. As we have previously described [19], these treatments alone result in robust AHR, combined with diverse inflammatory profiles, with neutrophilia following LPS treatment and lymphocytic inflammation following poly (I:C) treatment. These specific profiles indicate that our experimental model is well adjusted to mimicking bacterial and viral infections. LPS-induced neutrophilia is reminiscent of neutrophil accumulation as a pathological hallmark of bacterial lung disease [22], where hematopoietic cells and alveolar macrophages have been demonstrated to play a critical role in initiating LPS-induced neutrophil recruitment from the vascular space to the airspace [23]. It has also been shown that TLR4 plays a role in the pulmonary host defence against bacteria, as exemplified by *Haemophilius influenza* [24] and *Streptococcus pneumonia* [15]. Similarly, respiratory syncytial virus (RSV), which is a common cause of asthma exacerbations [3], particularly in childhood, induces a lymphocytic inflammatory response via TLR3 [14].

Using this model, we found that on a background of allergic inflammation, LPS and poly (I:C) challenge resulted in significantly heightened AHR, as compared to treatment of these ligands alone. More interestingly, LPS and poly (I:C) elevated AHR above the impact of OVA-treatment alone, exacerbating the effect of allergy. These changes mimic what is seen clinically, where respiratory symptoms in asthmatics are worsened following bacterial or viral infection [25], [26], alluding to a clear role for the innate immune TLRs in infection-induced exacerbation of asthma.

### Airway hyperresponsiveness does not correlate with inflammation in this model of exacerbation

The relationship between airway inflammation and AHR is complex, where former studies have shown a relationship between the severity of airway inflammation and AHR [27], whereas others have found no correlation [28], [29]. We previously described that AHR in response to TLR3 or TLR4 ligand stimulation is independent of inflammation, as AHR is similarly increased following LPS or poly (I:C) treatment, even when the inflammatory profiles induced by these microbial mimetics are diverse [19]. These findings are clearly recapitulated in this study. In this model, no significant change in neutrophillia in blood, lung tissue and BALF, or pro-inflammatory and neutrophil-associated cytokines in BALF was seen following LPS treatment in allergic animals. Similarly in these animals, cytokine release following poly (I:C) treatment was dramatically blunted. TH2 cytokines such as IL-13, which has previously been associated with AHR, showed no further upregulation with LPS treatment in OVA animals, further evidence to suggest a dissociation between AHR and inflammation. These results mirror findings from a study by Martin et al. [30], who showed that *Mycoplasma pneumoniae* increased bronchial hyperresponsiveness in an OVA-model of allergic lung inflammation, despite a significant decline in IFN-γ production.

Our results clearly demonstrate that TLR-induced AHR does not correlate with airway inflammation and thus posits the question as to how bacterial or viral components alter airway reactivity. It is unlikely that the effects of OVA and TLR ligand stimulation are simply additive, as poly (I:C) has previously been shown to have a greater effect than LPS on AHR when given alone, whereas the effect of LPS on AHR in OVA-treated animals is more prominent. TLR3 and TLR4 have been shown to be expressed in both mouse [31] and human [18] airway smooth muscle (ASM), suggesting a local effect of TLR ligands on ASM may be likely. Indeed, organ bath studies with isolated mouse trachea, which exclude the involvement of inflammatory cells, have shown an increased responsiveness to bradykinin following a 4-day pre-treatment with LPS or poly (I:C) [31]. It is unknown how or why, however, OVA-induced allergic inflammation could alter the responsiveness of the airway to TLR ligands. It has been well-described that ASM expresses functional TLRs and downstream signalling molecules [18], [31], [32], [33], which, upon activation, can result in cellular changes [31], [34], [35] that alter airway smooth muscle responsiveness. However, to our knowledge, no studies have investigated the impact of allergic inflammation on TLR expression or downstream signalling molecules on ASM. Further work is necessary to understand the mechanism by which OVA-treatment alters the airway responsiveness to TLR ligands.

### OVA-sensitisation and challenge alters the inflammatory response to TLR ligands

As noted, inflammation in response to LPS or poly (I:C) was significantly downregulated in animals with pre-established allergic lung inflammation. The rise in circulating neutrophils, as well as infiltrating neutrophils in lung tissue and BALF following LPS treatment in non-allergic animals was not apparent following the same treatment in allergic animals. This was associated with a similarly stunted production of cytokines associated with neutrophillic inflammation, including IL-17 [36], IL-12 [37], TNF-α, IL-6, IL-1α and MIP-1α. We further found that LPS treatment in non-allergic animals was associated with a rise in a particular population of neutrophils with low Ly6C expression, which was not apparent in allergic animals. To our knowledge, the role of heterogeneous surface expression in neutrophil activation and/or maturity has not been investigated. In monocytes, the level of Ly6C expression is often used as a descriptor for the maturity of the cell, where Ly6C^lo^ monocytes are deemed more mature [38]. Ly6C^lo^ neutrophils may similarly be in a more mature or differentiated state, which would correlate with the increase in cytokines and chemokines associated with neutrophil chemoattraction, as well as neutrophil activation found following LPS treatment in non-allergic, but not allergic, animals. LPS also failed to induce a CD8^+^ T-lymphocyte response in allergic animals. We similarly found a blunted anti-viral cytokine response following poly (I:C) treatment in allergic animals, where poly (I:C) failed to induce IFN-γ, IL-6, TNF-α, IL-12, RANTES and MIP-1α.

In this study, we could not clarify why the inflammatory response to microbial mimetics was blunted in allergic animals. However, it has been theorised that under conditions where the inflammatory response is skewed to a TH2 response, as in our allergic model, a TH1 response, characterised by a rise in IFN-γ and IL-12, cannot be fully established. A large degree of plasticity between TH1 and TH2 responses has been suggested [39], [40], but some suggest that this plasticity is only possible in early stages of differentiation [41], [42]. In a comprehensive study by Habibzay et al. [10], animals that had established allergic inflammation towards house dust mite (HDM), failed to respond to a *Streptococcus pneumoniae* infection, shown most prominently by reduced expression of neutrophil chemoattractants and a consequently a reduced neutrophillic response. It was suggested that this was in part due to alterations in the balance of TLR-regulatory proteins induced by the allergic environment, resulting in a failure to respond to TLR ligand stimulation. A similar study very recently showed that the immune response to HRV was significantly blunted in mice with HDM-induced chronic allergic inflammation, as seen by a failure to induce, among others, IL-12 and IFN-γ [43]. Indeed, in our study we found that inflammatory mediators associated with neutrophil chemoattraction and inflammation (RANTES, IL-1α, IL-17, IL-12, IL-6 and TNF-α) were significantly decreased in response to LPS or poly (I:C) in our allergic model. Surprisingly, however, no differences in KC, the mouse homologue of IL-8, in allergic animals treated with LPS versus non-allergic animals treated with LPS were detected at this time (Figure S3). Nevertheless, these studies together would suggest that in our model, an alteration TLR sensitivity may result in a blunted response to LPS or poly (I:C), which therefore results in the inability for these TLR ligands to promote an appropriate anti-microbial response. This further exaggerates the importance of TLR ligands in asthma exacerbation and may also explain the extended period of illness seen in asthmatics following a viral or bacterial infection.

It is of interest to mention kinetics when discussing inflammatory changes in this study, as a number of other studies have also looked at the effect of poly (I:C) and LPS on inflammation in models of OVA-dependent allergic inflammation. Compared to our study, where TLR agonists had no effect on TH2 cytokines or eosinophilia, Deuchs et al [44] has found that challenge with LPS and, in some cases poly (I:C), reduces levels of IL-4, IL-5 and eosinophilia in BALF when the TLR agonist is given 1 hr prior to each OVA challenge, between challenges or before all challenges. Similarly, whereas we found no increase in IL-6 or TNF-α in allergic animals treated with TLR agonists, as compared to PBS-treated allergic animals, TLR agonist treatment given 1 hr prior to OVA challenge results in a significant upregulation in these cytokines. In addition, Delayre-Orthez et al [45] has found that LPS reduces IL-4, IL-5 and eosinophil numbers when given during the sensitisation period, but has the opposite effect when given in conjunction with OVA challenges. Compared to our study however, these studies did not investigate how sensitised and TLR-treated animals differed from allergic, TLR-treated animals and we can therefore not be certain as to how kinetics of TLR and OVA challenge affect this parameter. Nevertheless, these studies are of interest as they clearly demonstrate that the kinetics of TLR and OVA challenge is vital to the inflammatory outcome and suggest, perhaps, that the time of infection in relation to the time of allergen exposure is key in predicting patient well-being. In future studies, it would be relevant to examine the kinetics of TLR and OVA challenge in our model in relation to shaping the inflammatory response, specifically the reduced response to TLR agonists in allergic animals. It would be particularly interesting to determine whether prior allergic inflammation leads to long-term dampening of an anti-microbial inflammatory response.

## Conclusion

Using the TLR ligands poly (I:C) and LPS, we showed in this study that bacterial or viral mimetics exacerbate AHR in a model of pre-established allergic lung inflammation, mirroring the impact of pathogenic exacerbation in human asthma and lending a role to TLRs in the exacerbation of respiratory symptoms. This was shown to be more profound in response to LPS, but nevertheless not absent in response to poly (I:C). Aggravated AHR was not associated with an increase in pro-inflammation, suggesting that AHR and inflammation are not correlated and implying that the impact of TLR ligands on AHR is a locally mediated mechanism. Rather, inflammation, particularly neutrophilia, was significantly blunted in allergic animals in response to LPS, the reason to which is unclear, but may reflect the extended duration of illness associated with bacterial or viral infection in asthmatics. Future studies should look to investigate the mechanism by which TLR ligands impact AHR, particularly following allergic inflammation, and also delineate how allergic inflammation impacts the inflammatory response to TLR ligands.

## Supporting Information

Figure S1 **Percent CD3^+^ lymphocytes in blood and tissue following TLR ligand challenge in OVA-sensitised mice.** All animals were sensitised i.p. with OVA/Al (OH)_3_ and subsequently challenged i.n. with PBS (grey bars) or OVA (black bars) (3 days) and PBS, LPS or Poly (I:C) (4 days). 24 hrs after the final challenge, blood and lung tissue were collected and percent of CD3^+^ lymphocytes were measured by flow cytometry. Data is represented as mean percent ± SEM. Data was analysed using a two-way ANOVA, followed by a Bonferroni multiple comparison post-test. *p<0.05; n = 6 per animals per group.
(PDF)

Figure S2 **CD4^+^CD3^+^ lymphocytes in blood and tissue following TLR ligand challenge in OVA-sensitised mice.** All animals were sensitised i.p. with OVA/Al (OH)_3_ and subsequently challenged i.n. with PBS (grey bars) or OVA (black bars) (3 days) and PBS, LPS or Poly (I:C) (4 days). 24 hrs after the final challenge, blood and lung tissue were collected and percent of CD4^+^ CD3^+^ lymphocytes were measured by flow cytometry. Data is represented as mean percent ± SEM. Data was analysed using a two-way ANOVA, followed by a Bonferroni multiple comparison post-test. n = 6 per animals per group.
(PDF)

Figure S3 **KC levels in BALF following LPS or poly (I:C) challenge in OVA-sensitised mice.** All animals were sensitised i.p. with OVA/Al (OH)_3_ and subsequently challenged i.n. with PBS or OVA (3 days) and PBS, LPS or Poly (I:C) (4 days). 24 hrs after the final challenge, BALF was extracted and KC levels were measured using the Cytokine Mouse 20-Plex Panel. Data is represented as mean ± SEM. Data was analysed using a two-way ANOVA, followed by a Bonferroni multiple comparison post-test **p<0.01, ***p<0.001. n = 6–14 animals per group.
(PDF)
