# Supplementary material for: Toll-Like Receptor Ligands LPS and Poly (I:C) Exacerbate Airway Hyperresponsiveness in a Model of Airway Allergy in Mice, Independently of Inflammation
Source: PLoS One. 2014 Aug 4;9(8):e104114. doi: 10.1371/journal.pone.0104114 (PMC4121312; doi:10.1371/journal.pone.0104114)
Supplement: Figure S2 — CD4+CD3+ lymphocytes in blood and tissue following TLR ligand challenge in OVA-sensitised mice. All animals were sensitised i.p. with OVA/Al (OH)3 and subsequently challenged i.n. with PBS (grey bars) or OVA (black bars) (3 days) and PBS, LPS or Poly (I:C) (4 days). 24 hrs after the final challenge, blood and lung tissue were collected and percent of CD4+ CD3+ lymphocytes were measured by flow cytometry. Data is represented as mean percent ± SEM. Data was analysed using a two-way ANOVA, followed by a Bonferroni multiple comparison post-test. n = 6 per animals per group. (PDF) [file pone.0104114.s002.pdf]

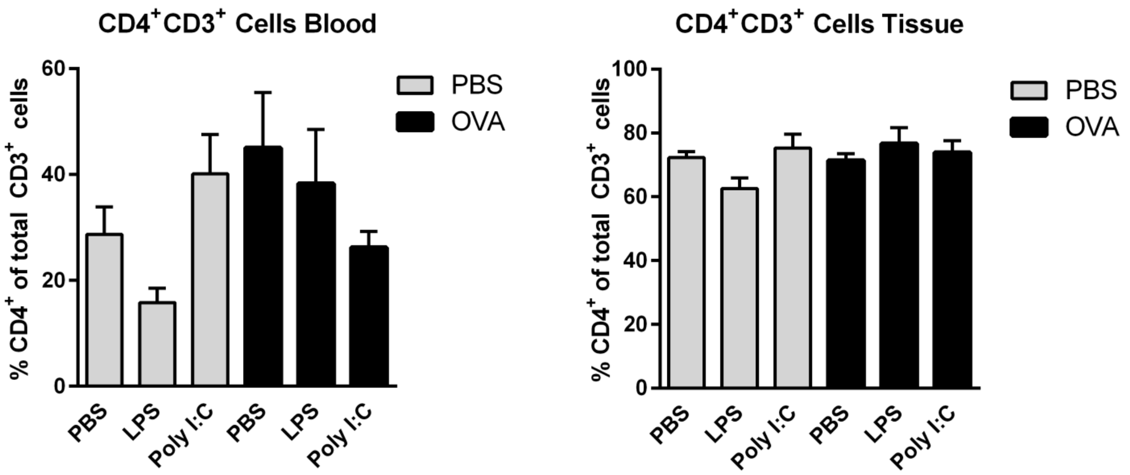

Figure S2 - $CD4^+CD3^+$ lymphocytes in blood and tissue following TLR ligand challenge in OVA-sensitised mice. All animals were sensitised i.p. with OVA/$Al(OH)_3$ and subsequently challenged i.n. with PBS (grey bars) or OVA (black bars) (3 days) and PBS, LPS or Poly(I:C) (4 days) . 24 hrs after the final challenge, blood and lung tissue were collected and percent of $CD4^+$ $CD3^+$ lymphocytes were measured by flow cytometry. Data is represented as mean percent ± SEM. Data was analysed using a two-way ANOVA, followed by a Bonferroni multiple comparison post-test. n=6 per animals per group.
