# Supplementary material for: Toll-Like Receptor Ligands LPS and Poly (I:C) Exacerbate Airway Hyperresponsiveness in a Model of Airway Allergy in Mice, Independently of Inflammation
Source: PLoS One. 2014 Aug 4;9(8):e104114. doi: 10.1371/journal.pone.0104114 (PMC4121312; doi:10.1371/journal.pone.0104114)
Supplement: Figure S3 — KC levels in BALF following LPS or poly (I:C) challenge in OVA-sensitised mice. All animals were sensitised i.p. with OVA/Al (OH)3 and subsequently challenged i.n. with PBS or OVA (3 days) and PBS, LPS or Poly (I:C) (4 days). 24 hrs after the final challenge, BALF was extracted and KC levels were measured using the Cytokine Mouse 20-Plex Panel. Data is represented as mean ± SEM. Data was analysed using a two-way ANOVA, followed by a Bonferroni multiple comparison post-test **p<0.01, ***p<0.001. n = 6–14 animals per group. (PDF) [file pone.0104114.s003.pdf]

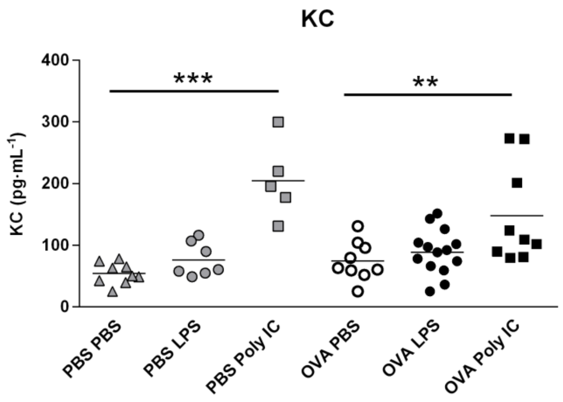

Figure S3 - KC levels in BALF following LPS or poly(I:C) challenge in OVA-sensitised mice. All animals were sensitised i.p. with OVA/$Al(OH)_3$ and subsequently challenged i.n. with PBS or OVA (3 days) and PBS, LPS or Poly(I:C) (4 days). 24 hrs after the final challenge, BALF was extracted and KC levels were measured using the Cytokine Mouse 20-Plex Panel. Data is represented as mean ± SEM. Data was analysed using a two-way ANOVA, followed by a Bonferroni multiple comparison post-test $^{**}p<0.01$, $^{***}p<0.001$. n=6-14 animals per group
